# Fabrication of New Hybrid Scaffolds for *in vivo* Perivascular Application to Treat Limb Ischemia

**DOI:** 10.3389/fcvm.2020.598890

**Published:** 2020-11-19

**Authors:** Michele Carrabba, Eva Jover, Marco Fagnano, Anita C. Thomas, Elisa Avolio, Thomas Richardson, Ben Carter, Giovanni Vozzi, Adam W. Perriman, Paolo Madeddu

**Affiliations:** ^1^Bristol Medical School, Translational Health Sciences, University of Bristol, Bristol, United Kingdom; ^2^School of Cellular and Molecular Medicine, University of Bristol, Bristol, United Kingdom; ^3^Research Centre ‘E. Piaggio', University of Pisa, Pisa, Italy; ^4^Dipartimento di Ingegneria dell'informazione, University of Pisa, Pisa, Italy

**Keywords:** tissue engineering, angiogenesis, adventitial pericytes, hybrid scaffold, biomaterials, bioprinting, electrospinning

## Abstract

Cell therapies are emerging as a new therapeutic frontier for the treatment of ischemic disease. However, femoral occlusions can be challenging environments for effective therapeutic cell delivery. In this study, cell-engineered hybrid scaffolds are implanted around the occluded femoral artery and the therapeutic benefit through the formation of new collateral arteries is investigated. First, it is reported the fabrication of different hybrid “hard-soft” 3D channel-shaped scaffolds comprising either poly(ε-caprolactone) (PCL) or polylactic-co-glycolic acid (PLGA) and electro-spun of gelatin (GL) nanofibers. Both PCL-GL and PLGA-GL scaffolds show anisotropic characteristics in mechanical tests and PLGA displays a greater rigidity and faster degradability in wet conditions. The resulting constructs are engineered using human adventitial pericytes (APCs) and both exhibit excellent biocompatibility. The 3D environment also induces expressional changes in APCs, conferring a more pronounced proangiogenic secretory profile. Bioprinting of alginate-pluronic gel (AG/PL), containing APCs and endothelial cells, completes the hybrid scaffold providing accurate spatial organization of the delivered cells. The scaffolds implantation around the mice occluded femoral artery shows that bioengineered PLGA hybrid scaffold outperforms the PCL counterpart accelerating limb blood flow recovery through the formation arterioles with diameters >50 μm, demonstrating the therapeutic potential in stimulating reparative angiogenesis.

## Introduction

Acute limb ischemia is the sudden loss of limb perfusion and is typically caused by an occluding embolus, *in situ* formation of a thrombus, trauma, or dissection of a peripheral artery. Chronic total occlusions of the femoral artery and implanted bypass grafts are common in patients with symptomatic peripheral artery disease (PAD). It is estimated more than 200 million people worldwide are affected by PAD (1). Approximately 12–20% of people over the age of 60 develop PAD with many developing critical limb ischemia (CLI), which is associated with a poor quality of life and a high risk of amputation and death (2). Artery occlusion represents a dramatic event that threatens limb viability and requires urgent evaluation and intervention (3). The early stage of the pathology is usually treated with pharmacological administration, whilst more acute forms of PAD are treated with surgical intervention, via insertion of hydrophilic wires to dissect through the intima or the media layers of the artery (4, 5). However, surgical treatments are subjected to durability issues related to the progression of the underlying pathology and the need for repetitive interventions (6, 7). In addition, in the most severe cases of CLI, patients are not suitable for surgical revascularization due to multiple occlusions and lack of autologous vessel replacement (8).

Approaches based on cell and gene therapy are in development to promote vascular repair and tissue reperfusion, stimulating reparative angiogenesis. Several studies have demonstrated that the transplanted cells performed their therapeutic action by a number of mechanisms including, direct incorporation into the host tissue, (9) activation and recruitment of resident stem cells, (10, 11) and by the release of pro-angiogenic factors, such as growth factors and micro-RNAs, able to activate the re-vascularization process (12–14). In the last few decades, cell-based therapies have led to several promising pre-clinical studies (10, 12, 15) and ultimately, clinical trials using injected bone marrow-derived and peripheral blood-derived hematopoietic cells have shown initial evidence of safety and therapeutic efficacy (7, 16). Despite the promising results, the low efficiency of cell retention in the ischemic area with a significant reduction of stem cells a short time after the injection (17, 18) and consequent reduction of therapeutic action represent the main weaknesses of this approach. Moreover, injected stem cells are prone to accumulation in tissue sinks, such as the lungs, liver and spleen (19).

Many researchers are now focusing on scaffold-based tissue engineering approaches to develop systems to achieve optimal delivery of the cell product to the arterial occlusion. Pericytes, EPCs and mesenchymal stem cells (MSCs) have shown the ability to encourage neovascularization when encapsulated in alginate or arginine-glycine-aspartic acid conjugated alginate micro-particles (13, 20–22). Positive outcomes in ischemic tissue of myocardial infarction were also shown by the use of injectable hydrogels, such as polyethylene-glycol (PEG), fibrin glue, chitosan hydrogel, and dextran-hyaluronic acid hydrogel when combined with BM-derived stem cells and MSCs (23–26). However, approaches involving a large number of synthetic microparticle-bound cells need to achieve a minimum beneficial effect that can disrupt the ischemic region, and lead to adverse clinical outcomes. Moreover, the inability to reproduce a supportive microenvironment with relevant mechanical properties limits the potential for hydrogel-based delivery systems.

Alternative methodologies use “hard” scaffolds as cell carriers, with the advantage of selecting the appropriate design of the structure according to the specific application. Indeed, the scaffold's material should attempt to match the topographic, mechanical, and bioresorption/remodeling features of the host tissue in order to stimulate the endogenous healing response. This approach offers the potential benefits of stimulating vasculogenesis both by delivering cells and by scaffold action directly (27, 28). Several natural and synthetic polymers have been explored and among those polycaprolactone (PCL) has been widely used. PCL nanofibers conjugated with fibronectin, and PCL 3D printed structures functionalized with gelatin (GL) nanofibers have been used to deliver MSCs (29) and adventitial pericytes (30) to infarcted myocardium and ischemic limb, respectively, to stimulate revascularization. Despite the increasing number of studies focused on using engineered scaffolds to treat ischemic tissues by stimulating spontaneous angiogenesis, the dominant mechanism of action is still largely unknown.

In this study, we show hybrid hard-soft scaffolds, comprised of microfabricated polymeric scaffolds with bioprinted hydrogel seeded with vascular cells, had beneficial pro-angiogenic effects in a murine model of LI. The novel hybrid scaffold, consisting of a 3D printed synthetic polymer [PCL or polylactic-co-glycolic acid (PLGA)] covered by a layer of electrospun GL nanofibers, were fabricated to recapitulate a rudimentary morphology and mechanical environment of the extracellular matrix (ECM) surrounding the femoral artery. In addition, the topography of the scaffold, enhanced by the cell-laden bioprinted gel, provided preferential growth direction for the seeded cells. Adventitial pericytes (APCs) isolated from saphenous veins were selected to functionalize the scaffold due to their ability to promote neovascularization or cell recruitment by secretion of paracrine factors (10, 14, 31). The scaffold was further functionalized with HUVECs to increase the therapeutic effect. Significantly, the cellularized hybrid scaffolds were highly biocompatible and promoted *in vitro* pro-angiogenic responses by the cells, with increased expression of VEGF, ANGPT-1 and FGF observed. The *in vivo* efficacy of this approach was investigated using a mouse model of LI, with the fabricated hybrid scaffolds placed around the occluded femoral artery. Here, the bioengineered PLGA hybrid scaffold outperformed the PCL counterpart by accelerating limb blood flow recovery and increasing the number of functional arterioles which supported scaffold resorption rate over matched mechanical properties as the dominate indicator of scaffold performance.

## Materials and Methods

### Cell Lines and Cultures

Studies using human cells were covered by Research Ethics Committee approvals (06/Q2001/197 and 11/2009) and complied with the principles stated in the 1964 Declaration of Helsinki and later amendments. All the subjects gave informed written consent for the experimental use of donated material (Supplementary Table 1). APCs were obtained from saphenous vein leftovers using immunomagnetic beads sorting and an expansion protocol described previously (31). The antigenic phenotype was evaluated by flow cytometry using a FACS Canto II flow cytometer and FACS Diva software (BD Biosciences). A combination of the following antibodies was employed: anti-CD44 (eBioscience), anti-CD-105 (Life Technologies) and anti-CD90 (BD biosciences). The purity of the cell preparation was confirmed, with >95% cells expressing the above markers. All *in vitro* and *in vivo* experiments were set up with APCs at passage 6. Commercially available human umbilical vein endothelial cells (HUVECs, Lonza cat#: CC-2517, lot:460587) were cultured at 37°C, 20% O_2_, 5% CO_2_ in complete Endothelial growth medium-2 (EGM-2, PromoCell) and used between passages 5 and 7.

### Materials and Reagents

PCL (Mn average 80,000), Pluronic® F-127 (PL), Sodium Alginate (AG) and gelatin from porcine skin (GL) were purchased from Sigma-Aldrich. 50:50 PLGA (Inherent Viscosity = 0.55–075 dL/g) was purchased from DURECT corporation (Cupertino, US). Gelatin crosslinking agent γ-glycidoxypropyl-trimethoxysilane (GPTMS) from Sigma-Aldrich. Endothelial basal medium-2 (EBM-2, cat# C-22211, Promocell) was used for *in vitro* specific assays, while complete EGM-2, consisting of EBM-2 supplemented with SupplementPack (cat#: C-39211, PromoCell), was used to culture APCs and HUVECs. Fetal bovine serum (FBS) was obtained from Hyclone (UT, USA). Phosphate buffer saline (PBS), penicillin and streptomycin were purchased from Gibco BRL, Invitrogen Corp., (Carlsbad, CA, USA).

### Hybrid Scaffold Preparation

#### Polymeric Scaffold Fabrication

The hybrid scaffold was fabricated using different techniques which followed a bottom-up approach aiming at mimicking the hierarchical organization of the natural extracellular matrix (ECM).

**Figure 1:**
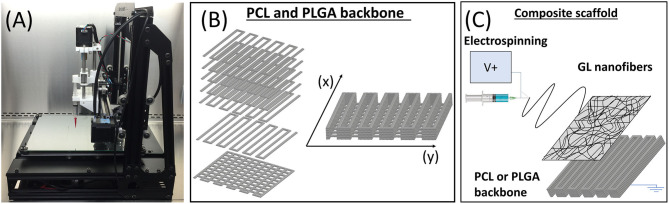
Schematic description of scaffold fabrication process. **(A)** Image of the customized piston-driven 3D printing system (MandleMax3, Maker's Tool Works, US); **(B)** 3D render of the designed pattern for channels structures of poly(ε-caprolactone) (PCL) and polylactic-co-glycolic acid (PLGA), highlighting the piling of various layers and the directions (x) and (y); **(C)** Schematic 3D representation showing the electrospinning of the gelatin (GL) nanofibers on the upper surface of the synthetic backbone of the composite scaffold (PCL or PLGA).

The fabrication of the scaffold followed the procedure reported in the previous work (30). In brief, the nanoscale structure of natural GL polymer was overlapped to a pre-existing matrix of synthetic material. In this study, PCL and PLGA were used as a synthetic backbone to generate two types of scaffolds with different physical properties. The synthetic matrices were manufactured with a customized piston-driven 3D printing system (MandleMax3, Maker's Tool Works, US) (Figure 1A), which allowed the extrusion of PCL and PLGA through a layer-by-layer deposition. PCL 10% (w v^−1^), and PLGA 15% (w v^−1^) were dissolved in chloroform, loaded in a glass syringe with a 32 gauge needle and extruded to generate a channel pattern. The channel pattern consisted of the deposition of a first layer with the shape of a grid, to avoid the collapse of the structure, followed by six layers of parallel lines, to form the walls of the channels (Figure 1B). A solution of Hydrolene® LTF/K (Ecopol S.p.A., Italy) [4% (w v^−1^) in distilled water] was used as support material during the extrusion of the polymeric solutions. The composite scaffolds were completed by the electrospinning of GL nanofibers on top of the synthetic structures to improve the biomimetic and adhesive features of the scaffold (Figure 1C). After dissolving porcine GL in acetic acid-water solution (ratio of 60:40) at the concentration of 15% (w v^−1^), the crosslinking agent GPTMS was added at the concentration of 3% (v v^−1^) and stirred for 1 h before the use of the solution. The GL solution was then electrospun using an Electrospinning Station (Nadetech®, Navarra Spain), setting parameters to the following values: 25 kV (Voltage), 20 cm (distance from the collector) and 0.2 ml h^−1^ (flow rate).

#### Mechanical Property of the Scaffold

Mechanical characterization of the scaffold was performed using Instron 3343 (Illinois Tool Works Inc., US). PCL- and PLGA-based scaffolds, fabricated with the dimension of 12 × 12 mm wide specifically for this test, were clamped with pneumatic grips with an initial displacement of 5 mm. The uniaxial test machine was set with a steady deformation speed of [(0.006^*^ *l*_0_) min^−1^]. Polymeric structures were tested in both axial (x) and longitudinal direction (y). In addition, mechanical properties were further evaluated in relation to the degradation rate. PCL- and PLGA-based scaffolds were conditioned with EBM-2 and incubated at 37°C, mimicking the cell culture environment, and uniaxial tests were performed at specific time-points [0, 2, 4, 7, 10 days (*n* = 3 each time-point)].

### Scaffold Cellularization

#### APC Seeding on Scaffold

PCL- and PLGA-based scaffolds of the dimension of 6 × 6 mm wide was prepared following an established sterilization protocol. Scaffolds underwent sequential washes with 70% ethanol and PBS and then exposed to UV light for 15 min. The polymeric structures were then washed with PBS and conditioned with EGM-2 culture medium for 1 h. APCs, at passage 6, were then seeded at a density of 6,000 cell cm^−2^ onto scaffolds and cultured for 5 days, with medium changed on day 3.

#### Functional Assays

The cell behavior after seeding and culturing on different polymeric materials was assessed by evaluating density, viability, and proliferation. APCs were labeled with the fluorescent marker 1,1′-dioctadecyl-3,3,3′,3′-tetramethylindocarbocyanine perchlorate (Dil) (Cell-TrackerTM CM-DiI, Molecular Probes, Leiden, Netherlands) and seeded onto the scaffold as described above. After 5 days of incubation, cellularized scaffolds were washed with PBS, fixed with PFA 4%, then counterstained with DAPI, mounted with antifade mounting medium.

The viability of the APCs seeded onto PCL- and PLGA-based scaffold was assessed using the viability/cytotoxicity assay kit (Biotium Inc, US). Five days after seeding, the culture medium was removed, the scaffold washed with PBS and incubated for 30 min with a solution of Calcein [1:2000], EthD-III [1:500] and Hoechst [1:100] in serum-free medium at 37°C, 5% CO^2^. Viable cells were identified using fluorescence microscopy. The ability of cells to proliferate once seeded onto scaffolds was evaluated by Click-iT® EdU Assay (Life Technologies, UK). Fluorescent images of the scaffolds were obtained using a Zeiss Fluorescent Microscope (Zeiss Axio observer Z1, Zeiss) and number of cells quantified using Image-Pro Plus software. All the functional assays in this study were performed on three cell lines, in technical triplicates.

#### Reverse Transcriptase-Polymerase Chain Reaction (RT-PCR)

Total RNA was isolated from APC-bioengineered PCL and PLGA scaffolds by a standardized phenol-chloroform protocol combining QIAzol lysis and miRNeasy mini kit (QIAGEN, Germany), following manufacturer's instructions.

Briefly, PCL- and PLGA-based scaffolds seeded with APCs for 5 days were washed once in PBS to remove non-adhered cells. Scaffolds were then collected from the culture plate, lysed in tubes with 1 ml QIAzol l, and stored on ice. The tubes were centrifuged to separate debris of polymeric structures and the supernatant was transferred to clean tubes. Cell monolayers seeded in culture petri dishes, used as control [hereafter referred to as bi-dimensional (2D) counterparts], were washed with PBS, and incubated with QIAzol; then, cell lysates were collected in tubes. Scaffold derived debris were removed by centrifuging at 10,000 g, 3 min, 4°C before chloroform separation of organic and inorganic phases. Chloroform was added to both cell lysates from scaffolds (3D) and 2D counterparts, followed by centrifugation at 12,000 × g for 15 min at 4°C.

Resulting total RNA was reverse-transcribed into single-stranded cDNA using a High Capacity RNA-to-cDNA Kit (Life Technologies, UK) (100 ng RNA), or using specific Taqman microRNA assay primers with a TaqMan® MicroRNA Reverse Transcription Kit (10 ng) for the assessment of microRNA expression (both from Life Technologies). Quantitative real-time PCR (qPCR) of first-strand cDNA was performed using TaqMan Fast Universal PCR Master Mix or SYBR Green PCR (both from Life Technologies, UK) as appropriate into a QuantStudio 6 Flex (Thermo Fisher Scientific). Targeted genes include markers for apoptosis, angiogenesis, pericyte profile and myofibroblast transformation: *BAX* (Hs00180269_m1), *BCL2* (Hs04986394_s1), *PDGFRB* (Hs01019589_m1), *ANG-1* (Hs00375822_m1), *VEGFA* (hs00900055_m1), *BACH1* (Hs00230917_m1), *FGF* (Hs01092738_m1) (all of them TaqMan® probes, Applied Biosystems); *ACTA2/SMA* (QT00088102, Hs_ACTA2_1_SG), *TGLN/SM22A* (QT00072247, Hs_TAGLN_1_SG), *COL1A1* (QT00037793, Hs_COL1A1_1_SG), *MYOC* (QT00068327, Hs_MYOC_1_SG), and *MYH11* (QT00069391, Hs_MYH11_1_SG) (all of them. QuantiTect Primer Assay-QIAGEN for SYBR Green applications).

MicroRNA profile preservation was additionally assessed with specific miRNA assay probes has-miR-132-3p (assay no. 000457), hsa-miR-532-5p (assay no. 001518), and hsa-miR-210-3p (assay no. 000512). miRNA expression was normalized to U6 snRNA (assay no.001973) (all of them TaqMan® probes, Applied Biosystems).

Relative mRNA expression was calculated using the 2^−ΔΔ*ct*^ method (Livack method) and expressed as fold-change compared to 2D control counterparts. All experiments were performed on three biological replicates and assessed in technical triplicates.

#### Enzyme-Linked Immunosorbent Assays (ELISA)

Cell conditioned media (CCM) from APC-bioengineered scaffolds (3D) or 2D control counterparts was collected and assayed for angiopoietin-1 (ANG-1) and vascular endothelial growth factor-A (VEGFA) using specific sandwich ELISA (DuoSet ELISA, R&D Systems). In brief, after 3 days in culture, EGM-2 growing media was replaced with all control and experimental conditions with fresh growth factor-depleted EBM-2. Cells were kept for 48 h more at 37°C, 5% CO_2_, 21% O_2_ and CCM were collected and centrifuged at 10,000 g, 3 min, 4°C to remove cell debris. CCMs were kept at −80°C until batch analysis. Data are shown as fold change by comparing to 2D control counterparts to assess for biological significance of our findings and avoid the effect of donor heterogeneity.

### Final Hybrid Scaffold Preparation

#### Hydrogel Preparation: Sodium Alginate/Pluronic Based Gel

AG and PL solutions were produced following the protocol described by Armstrong et al. (32). Briefly, the final hydrogel working solution was produced by combining solutions of PL and AG to achieve a final gel of 13% (w v^−1^) PL and 6% (w v^−1^) AG in serum-free DMEM (Gibco Life Technologies™). The gel was then crosslinked with CaCl_2_ to achieve higher water resistance.

#### Hydrogel Printing Characterization

Hydrogel printability and biocompatibility were further assessed before the final assembly. The extrusion of the cell-laden hydrogel was also performed with the piston-driven 3D printing system. A pattern of 12 × 12 mm grid was chosen to evaluate the gel printability. Solutions of EBM-2 with CaCl_2_ at different concentrations were used to assess the shape maintenance of the structures after printing, followed by the analysis of cytotoxicity effect of the crosslinking agent. Both APCs and HUVECs were tested. The cell-laden gel solution of AG/PL gel, [13% (w v^−1^) PL and 6% (w v^−1^)] with cells were loaded into a 1 ml sterile and disposable syringe, fixed to the bioprinter holder, with subsequent hydrogel printing onto a sterile coverslip. For these tests, cell concentration was fixed at 1 million·ml^−1^; while, for the final scaffold, concentration was increased up to 4 million·ml^−1^. The extruded structures were incubated with CaCl_2_-EBM-2 solutions (5, 10, 20, 30, 50, and 100 mM) for 10 min at 37°C and 5% CO_2_ for the first crosslinking phase. The gels were washed with PBS and incubated a second time with 5 mM CaCl_2_ solution at 37°C and 5% CO_2_. After additional 24 h, the CaCal_2_-medium was changed with complete EGM-2 and incubated for 24 h. At the end of this period, fluorescent images acquired with Zeiss Fluorescent Microscope were used to evaluate the shape of the extruded pattern and viability of cells was assessed using a dedicated kit (Biotium Inc, US). Percentages of viable cells were quantified with Image-Pro Plus.

#### Cell-Laden Gel Patterning

PCL-based and PLGA-based scaffolds coated with GL nanofibers crosslinked with GPTMS were cellularized with APCs, as described above. After a culture period of 5 days, scaffolds were used as a substrate to extrude a patterned layer of the cell-laden gel. Co-culture of APCs and HUVECs [1:4] was loaded in AG/PL gel [13% (w v^−1^) PL and 6% (w v^−1^)] with a total cell concentration of 4 million ml^−1^, gently mixed to homogenize the solution and then transferred into a 5 ml syringe with a 27 gauge needle for the bioprinting process. The cellularized scaffold was quickly placed on the heated plate of the Bioprinter and the extrusion was activated. The cell-laden gel was deposited on the scaffold with the pattern of lines parallel to the (*x*) direction of the scaffolds. At the end of the extrusion, the scaffold was incubated with 100 mM CaCl_2_-EBM-2 solution for 10 min. Then, the solution was removed and 5 mM solution of CaCl_2_-EGM2 (supplemented with 1% penicillin/streptomycin) was added. This established protocol was used to prepare hybrid scaffold (PCL-GL + AG/PL) and PLGA-GL+AG/PL) for *in vivo* implantation.

### *In vivo* Angiogenesis

#### Animal Model

Experiments involving live animals were performed in accordance with the *Guide for the Care and Use of Laboratory Animals* (The Institute of Laboratory Animal Resources, 1996) under British Home Office PPL 30/3373, after Ethical approval from the University of Bristol. Data were reported according to the ARRIVE guidelines. Male C57Bl/6J mice (8 weeks old; Charles River, UK) underwent unilateral femoral artery ligation under isoflurane anesthesia. The proximal and the distal end of the femoral artery were occluded using 6-0 silk, and the portion of the artery between the ligations was electro-coagulated. Group size was calculated to detect a 20% difference in the primary endpoint between groups with *a* = 0.05 and a power of 80%. Mice were randomly assigned to five experimental groups (*n* = 10 per group) described as follows: group I was given no treatment (vehicle); group II was implanted with PCLGL + AG/PL without cells; group III with PLGA-GL + AG/PL without cells; group IV with PCL-GL + AG/PL with cell; and group V with PLGA-GL + AG/PL with cells. Scaffolds were properly dimensioned in 3 × 3 mm squares for the *in vivo* application. During the implantation procedure, the scaffolds were positioned between the ligations and wrapped around the occluded femoral artery, with the PCL or PLGA in contact with the artery and the gel with or without cells exposed to the perivascular tissues. After being given Vetergesic analgesia, the animals were allowed to recover. The mice were given standard chow and water *ad libitum* and inspected regularly for any change in clinical signs.

The primary endpoints were blood flow recovery and vascular density. The ischemic foot was sequentially monitored by color laser Doppler at 0, 3, 7, 14, and 21 days after induction of ischemia (33). The recovery was assessed by comparing the ratio of flow in the ischemic and contralateral legs. Mice were killed under terminal anesthesia on day 21. The adductor muscles and the perivascular area (including the femoral artery and scaffold) were excised intact, fixed with PFA (4% w v^−1^ in PBS) overnight, and then embedded in optimal cutting temperature (OCT) medium. Samples were sectioned at a thickness of 5–7 μm using a Cryotome (LEICA RM2235, Germany).

#### Immunohistochemistry Staining

For analysis of vascularization, sections of adductor muscle and the perivascular area were incubated overnight at 4°C with primary α-SMA-Cy3 (c6198, Sigma, UK), to identify VSMCs, and Alexa 488-conjugated isolectin B4 (Life Technologies, UK) to identify ECs. Streptavidin-Alexa 488 secondary antibody. Sections were counterstained with DAPI (30 nM), to identify nuclei, and coverslips mounted using antifade mounting medium.

#### Statistical Analysis

Continuous variables distribution was assessed by Kolmogorov–Smirnov Z normality test and Shapiro Wilk test and are shown as mean ± standard error of the mean (SEM) or standard deviation (SD) or as median (IQR), depending on the sample distribution. Continuous variables normally distributed were compared using the Student's *t*-test (two-group comparison) or one-way analysis of variance followed by Tukey PostHoc analysis (ANOVA; for multiple group comparisons), as appropriate. Two-way ANOVA analysis was used to compare the mean differences between groups in the animal model (two categorical and one continuous variable) followed by pair-wise comparison using the Holm-Sidak method. Non-parametric tests, including the Mann–Whitney U test or the Kruskal-Wallis test, were used for data not normally distributed. A *P*-value < 0.05 was considered statistically significant. Analyses were performed using GraphPad Prism 8.0 statistical software.

## Results

### Feasibility of Composite Polymeric Scaffold Production

In order to elucidate the *in vitro* and *in vivo* responses to scaffold biomaterials, which differ systematically in mechanical properties and resorption rate, we compared two synthetic polymers, PCL and PLGA. The hybrid hard-soft scaffolds were fabricated using an adaption of a bottom-up approach with the aim being to mimic the hierarchical organization of natural ECM (30). In brief, the nanoscale structure of natural GL polymer was overlapped on a pre-existing matrix of synthetic material. In this study, PCL and PLGA were used as the synthetic backbone to generate two types of scaffolds with different physical properties. The synthetic matrices were manufactured using a customized piston-driven 3D printing system (MandleMax3, Maker's Tool Works, US), which allowed the extrusion of PCL or PLGA via a layer-by-layer deposition.

The customized piston-driven printing system (Figure 1A) allowed the extrusion of PCL (10% w v^−1^) or PLGA (15% w v^−1^) polymer solutions, following with high resolution the designed pattern of the channel (Figure 1B). Assessment by optical microscopy showed the full thickness of the scaffolds was 70 ± 10 μm and line width was 121 ± 15 μm for PCL and 127 ± 23 μm for PLGA, respectively (Figure 1C).

**Figure 2:**
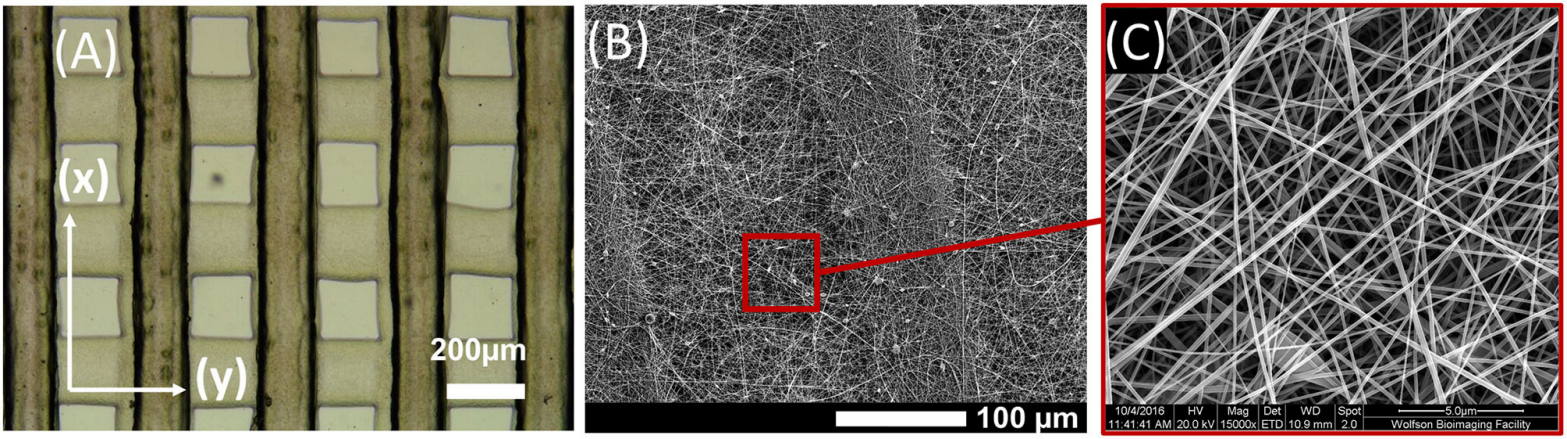
Fabricated multi-material scaffold. **(A)** Representative image of channel structure; **(B)** Representative SEM picture of the composite scaffold formed by gelatin (GL) nanofibers covering polylactic-co-glycolic acid (PLGA) cannels structure; **(C)** Representative SEM picture of the GL nanofibers.

The synthetic backbone of PCL or PLGA was then covered by electrospinning GL nanofibers directly onto the surface (Figure 2A). GL nanofibers were then crosslinked with GPTMS 3% (v v^−1^) to improve durability in wet conditions. The successful assembling of the PCL-GL or PLGA-GL multi-material scaffolds was assessed by SEM (Figure 2B). The mat of GL nanofibers was uniformly distributed and adherent to the upper surface of the synthetic materials. Additionally, the GL nanofibers were randomly oriented and had an average diameter of 90 ± 18 nm and a pore size 290 ± 18 nm (Figure 2C).

### Material Composition Confers Different Mechanical Properties to the Scaffolds

**Figure 3:**
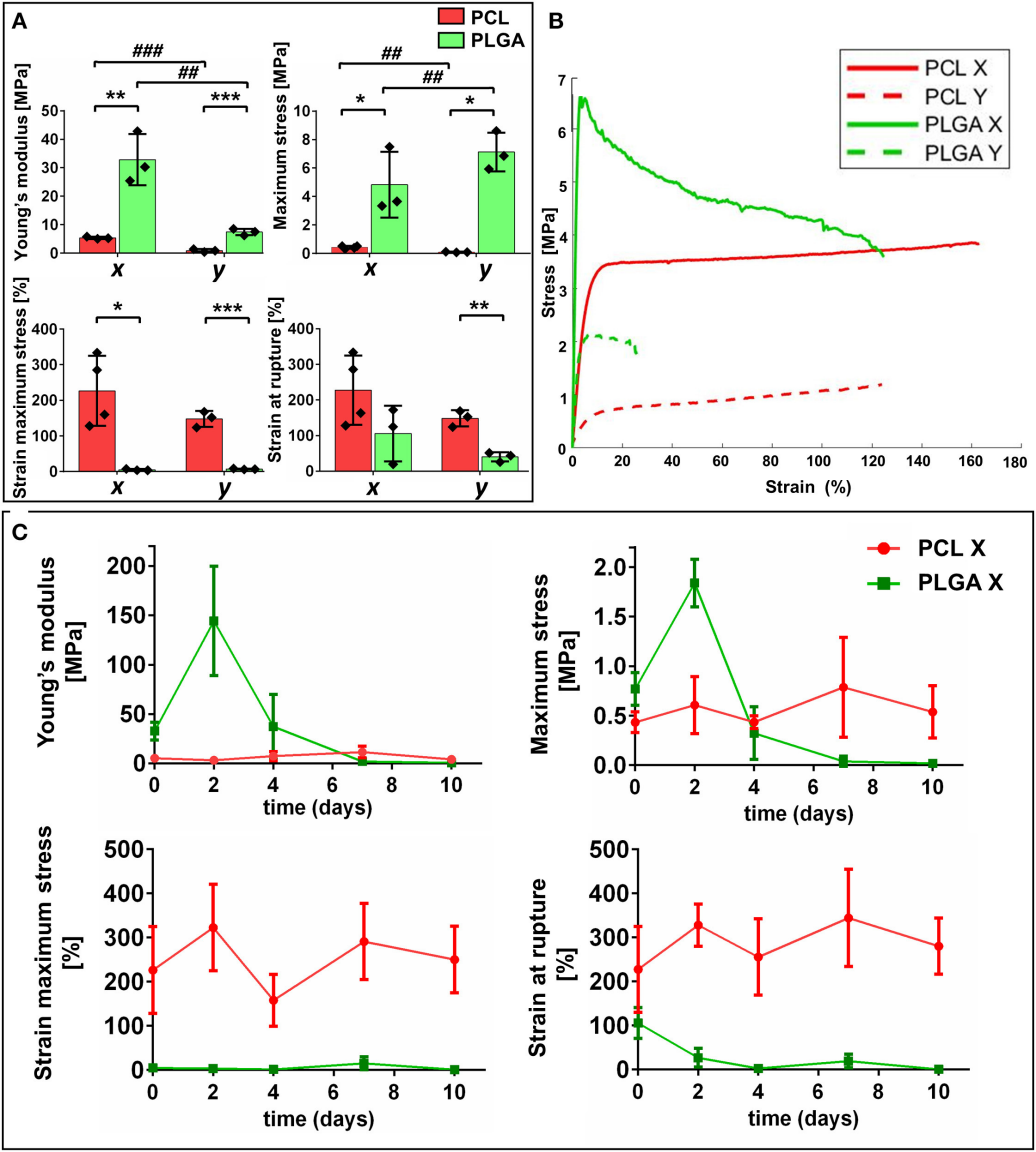
Mechanical characterization of the scaffolds. **(A)** Bar graphs summarize results of mechanical tests on poly(ε-caprolactone) (PCL) and polylactic-co-glycolic acid (PLGA) structures namely: Young's modulus, maximum stress, strain at maximum stress and strain at rupture. Values are means ± SD, *N* = 3 biological replicates, each one with 3 technical replicates. *P < 0.05, **P < 0.01, ***P < 0.001, vs. PCL-scaffold within same direction of testing [(x) or (y)]; ^##^*P* < 0.01, ^###^*P* < 0.001 vs. the (x) direction of the corresponding group (PCL or PLGA). **(B)** Stress–strain curves of synthetic structures (PCL and PLGA) in both directions (x and y). **(C)** Graphs showing mechanical features of PCL and PLGA scaffolds at different timepoints.

Mechanical tests were performed to evaluate the properties of the different biomaterials, the effect of the geometry and the response to incubation in wet conditions over time. The data from mechanical testing are reported in Figure 3A and Table 1. Scaffolds made of PCL had different values in the axial direction (*x*) compared with the longitudinal direction (*y*), namely higher values of Young's modulus [(*x*): 5.37 ± 0.37 vs. (*y*): 0.91 ± 0.51 MPa, *P* < 0.001] and maximum stress [(*x*): 0.43 ± 0.12 vs. (*y*): 0.09 ± 0.019 MPa, *P* < 0.01]. PLGA scaffolds followed similar patterns for both Young's modulus [(*x*): 32.86 ± 8.9 vs. (*y*): 7.44 ± 1.14 MPa, *P* < 0.01] and maximum stress [(*x*): 0.77 ± 0.16 vs. (*y*): 0.23 ± 0.07 MPa, *P* < 0.01]. These results indicate the two constructs have anisotropic mechanical characteristics.

**Table 1:** Mechanical properties of PCL and PLGA scaffold analyzed in both (x) and (y) direction.

	**Young's modulus (MPa)**	**Maximum stress (MPa)**	**Strain at maximum stress (%)**	**Strain at failure (%)**
PCL Ch-X	5.37 ± 0.40	0.43 ± 0.10	226.5 ± 98.5	230.7 ± 99
PCL Ch-Y	0.91 ± 0.51^###^	0.09 ± 0.019^##^	147.9 ± 22.3	148 ± 23.6
PLGA Ch-X	32.86 ± 8.9^**^	0.77 ± 0.16^*^	4.82 ± 2.3^*^	105.7 ± 70
PLGA Ch-Y	7.44 ± 1.14^***^, ^*##*^	0.23 ± 0.07^*^, ^*##*^	7.13 ± 1.35^***^	40.4 ± 13^**^

*Values are means ± SD, N = 3 biological replicates, each one with 3 technical replicates*.

* P < 0.05,

** P < 0.01,

*** P < 0.001, vs. PCL-scaffold within same direction of testing (Ch-X or Ch-Y);

## P < 0.01,

### *P < 0.001 vs. the Ch-X direction of the corresponding group (PCL or PLGA)*.

Comparing the PLGA and PCL structures, the former had significantly higher values of Young's modulus [(*x*): *P* < 0.01 and (*y*)*: P* < 0.001] and maximum stress (*P* < 0.05). Conversely, PCL showed higher values of strain at maximum stress [(*x*): *P* < 0.05 and (*x*): *P* < 0.001] and strain at rupture (*P* < 0.01). This data confirms the higher rigidity of the PLGA structure, as illustrated by the stress-strain curves in Figure 3B.

We next assessed the changes in mechanical features during incubation of the structure in EBM-2 for up to 10 days (Figure 3C). PCL showed resistance to degradation, its properties remaining steady until the end of the observational period. In contrast, the PLGA structure showed fast degradation, resulting in a drastic decrease of Young's modulus, maximum stress and strain at rupture from day 4 of incubation.

### Scaffold Cellularization With APCs and Biocompatibility Assessment

**Figure 4:**
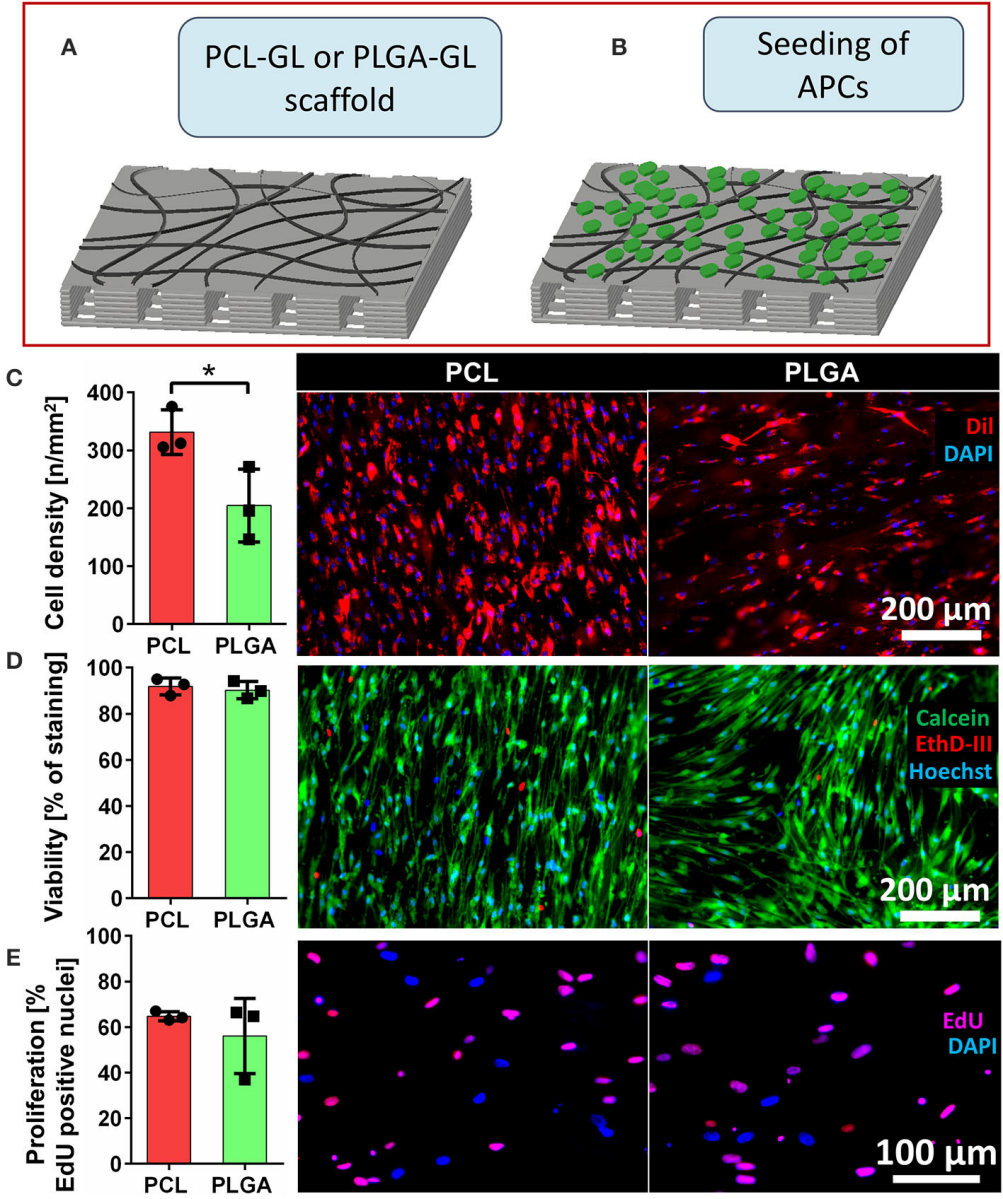
*In vitro* cellularization of scaffolds and functional assessment. **(A,B)** Schematic representation of the Hybrid scaffold preparation following the specific steps: **(A)** step a: fabrication of the composite scaffold formed by poly(ε-caprolactone (PCL) or polylactic-co-glycolic acid (PLGA) and gelatin (GL) nanofibers, named, respectively, PCL-GL and PLGA-GL; **(B)** step b: seeding with adventitial pericytes (APCs) and culturing for 5 days; **(C)** Bar graph of cell density and representative fluorescent microscopy images of PCL and PLGA scaffolds seeded with APCs. Nuclei are stained with DAPI (blue) and cell bodies are labeled with Dil (red). **(D)** Bar graph of cell viability and representative images of APCs: viable cells stained with calcein (green), dead cells with EthD-III (red) and all the nuclei with Hoechst (blue) for assessment of cell viability. **(E)** Bar graph of cell proliferation and representative images of APCs on scaffolds [proliferating cells stained by EdU (pink) and not proliferating ones with DAPI (blue)]. Values are means ± SD, *N* = 3 biological replicates, each one with 3 technical replicates. *P < 0.05, vs. PLGA-scaffold.

Having achieved a robust production protocol for the PCL-GL and PLGA-GL composite scaffolds, we next performed cellularization through two stages. The first stage consisted of APC seeding by pipette deposition onto the scaffold surface, and the evaluation of the *in vitro* interaction of the cells with the different biomaterials (Figures 4A,B). Cell monolayers seeded in culture petri dishes were used as control [hereafter referred to as bi-dimensional (2D) counterparts].

Five days after the seeding, cell density, viability and proliferation were evaluated. Overall, the results of the assays indicated good biocompatibility for both the PCL-GL and PLGA-GL scaffolds. Fluorescent microscopy confirmed the homogeneous growth of APCs on the scaffold surfaces, with PCL scaffolds having more adherent cells (*P* < 0.05) (Figure 4C) than PLGA scaffolds.

APC viability was high with both materials (90 ± 3% for PLGA and 92 ± 2% for PCL scaffold) (Figure 4D). These data were further validated at the molecular level by measuring the mRNA expression of pro-apoptotic *BAX* and pro-survival *BCL2* genes. The APC-bioengineered scaffolds showed a *BAX/BCL2* ratio superior to 2D control counterparts, but the difference did not reach statistical significance (PCL: 2.48 ± 0.58-fold change vs. 2D; PLGA: 1.47 ± 0.19-fold change vs. 2D). Cell proliferation was observed on both PCL and PLGA-based scaffolds, with 61 ± 4% and 56 ± 17% of cells showing Edu-positive nuclei, respectively (Figure 4E). Despite a certain degree of directionality given by the macro-domain of the polymeric scaffolds, APCs tended to rearrange in random directions.

### Culture in Scaffolds Modifies the Expressional Profile of APCs

**Figure 5:**
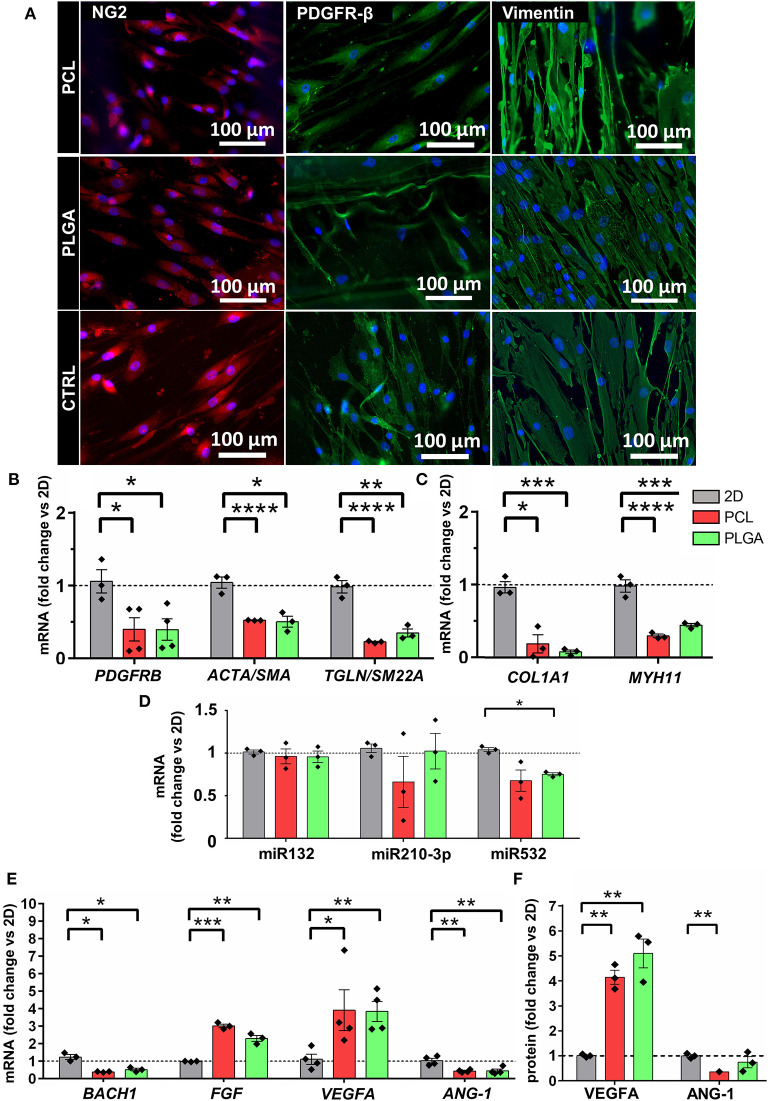
Effect of 3D culture on poly(ε-caprolactone) with gelatin nanofibers (PCL-GL) and polylactic-co-glycolic acid with gelatin nanofibers (PLGA-GL) on APCs. Bar graphs show the average of 3 biological replicates comparing the 3D conditions to the 2D monolayer culture on petri dish. **(A)** Representative fluorescence images of specific phenotype markers (NG2, PDGFR-β and Vimentin) in APCs seeded, respectively, on PCL- and PLGA-based scaffolds; images showed that all the cells were positive for the indicated markers. **(B,C)** Expression of differentiation (PDGFRB, ACTA2/SMA, TGLN/SM22A, COL1A1, and MYH11) molecules. **(D)** Expression of angiogenic miRs: miR132, miR210-3p, and miR532. **(E)** BACH1, FGF, VEGFA, and ANG-1 molecules. **(F)** Bar graph shows the secreted of VEGFA and ANG-1. *p < 0.05, **p < 0.01, and ***p < 0.001 vs. 2D. Values are means ± SE, *N* = 3 biological replicates, each one with 3 technical replicates. ****p < 0.0001.

In a parallel experiment, we investigated if PCL-GL or PLGA-GL scaffolds impair the characteristic antigenic and proangiogenic profile of APC. After 5 days of culture on the scaffolds, immunocytochemistry (ICC) was used to show APC protein expression of PDGFR-β, NG2 and vimentin markers (Figure 5A). Additionally, RNA was extracted and used to analyze the transcriptional signature of APCs by qPCR, using 2D control counterparts to calculate the relative mRNA expression. *PDGFRB*, a marker shared by pericytes and VSMC, was reduced in 3D conditions (PCL: 0.40 ± 0.16-fold change vs. 2D, *P* < 0.05; PLGA: 0.39 ± 0.15-fold change vs. 2D, *P* < 0.05). The mRNA levels of *ACTA2/SMA* (VSMC and myofibroblast marker), *TGLN/SM22A* (an early marker of smooth muscle differentiation), *COL1A1* (mainly expressed by myofibroblasts and active VSMC) and *MYH11* (a major VSMC contractile protein) were significantly down-regulated, thus suggesting the 3D environment affected the expression of genes that characterize the functional transition of pericytes toward myo-fibroblastic cells (Figures 5B,C). No differences were seen with respect to the mRNA expression of transcription factors *GATA4, SOX2*, and *NANOG*, which we have previously shown to be associated with the progenitor-like profile of human APCs (data not shown) (31).

Furthermore, previous studies have shown that APCs express several angiogenesis-related microRNAs (miRs) and transcription factors (34–36). The expression of miR132-3p and miR210-3p was similar in APCs embedded in PCL or PLGA compared to 2D control counterparts. However, miR532-5p was found to be down-regulated in APCs under 3D conditions, showing a significant difference vs. 2D for PLGA-embedded cells (*P* < 0.05) (Figure 5D). Expression of *BACH1*, a transcription factor which is a negative regulator of *ANGPT1* and heme-oxygenase-1, was downregulated in APC seeded on both polymers (PCL: 0.39 ± 0.03-fold change vs. 2D, *P* < 0.05; PLGA: 0.52 ± 0.13-fold change vs. 2D, *P* < 0.05) (Figure 5E). Interestingly, the three-dimensionality of the scaffold was associated with an increased expression of the arteriogenic factor *FGF* by APCs (PCL *P* < 0.001 vs. 2D and PLGA *P* < 0.01 vs. 2D). Moreover, APCs showed an upregulation of *VEGFA* mRNA in both PCL (3.92 ± 1.16-fold change, *P* < 0.05 vs. 2D) and PLGA scaffolds (3.85 ± 0.57-fold change, *P* < 0.01 vs. 2D). However, *ANGPT1* expression was significantly downregulated in cells seeded on the 3D structures (PCL: 0.44 ± 0.04-fold change vs. 2D, *P* < 0.01; PLGA: 0.45 ± 0.1-fold change vs. 2D, *P* < 0.01).

The expression of VEGFA and ANGPT1 were further verified by assaying the APC-derived secretome (Figure 5F). VEGFA was found to be up-regulated in both PCL (4.11 ± 0.53-fold change vs. 2D, *P* < 0.01; or 6.54 ± 1.06-fold change vs. 2D) and PLGA (5.07 ± 0.99-fold change vs. 2D, *P* < 0.01; or 7.33 ± 0.21-fold change vs. 2D). As per the qPCR results, ANGPT1 levels were reduced in PCL (0.36 ± 0.02-fold change vs. 2D, *P* < 0.01) but not in PLGA (*P* = 0.441). Altogether, the data suggest that 3D culture at this timepoint confers APCs with a pro-angiogenic growth profile, where induction cues (FGF/VEGFA) prevail over factors involved in stabilization (ANGPT1).

### Characterization of Structure Fidelity of Sodium Alginate-Pluronic Gel and Evaluation of Cell Viability

**Figure 6:**
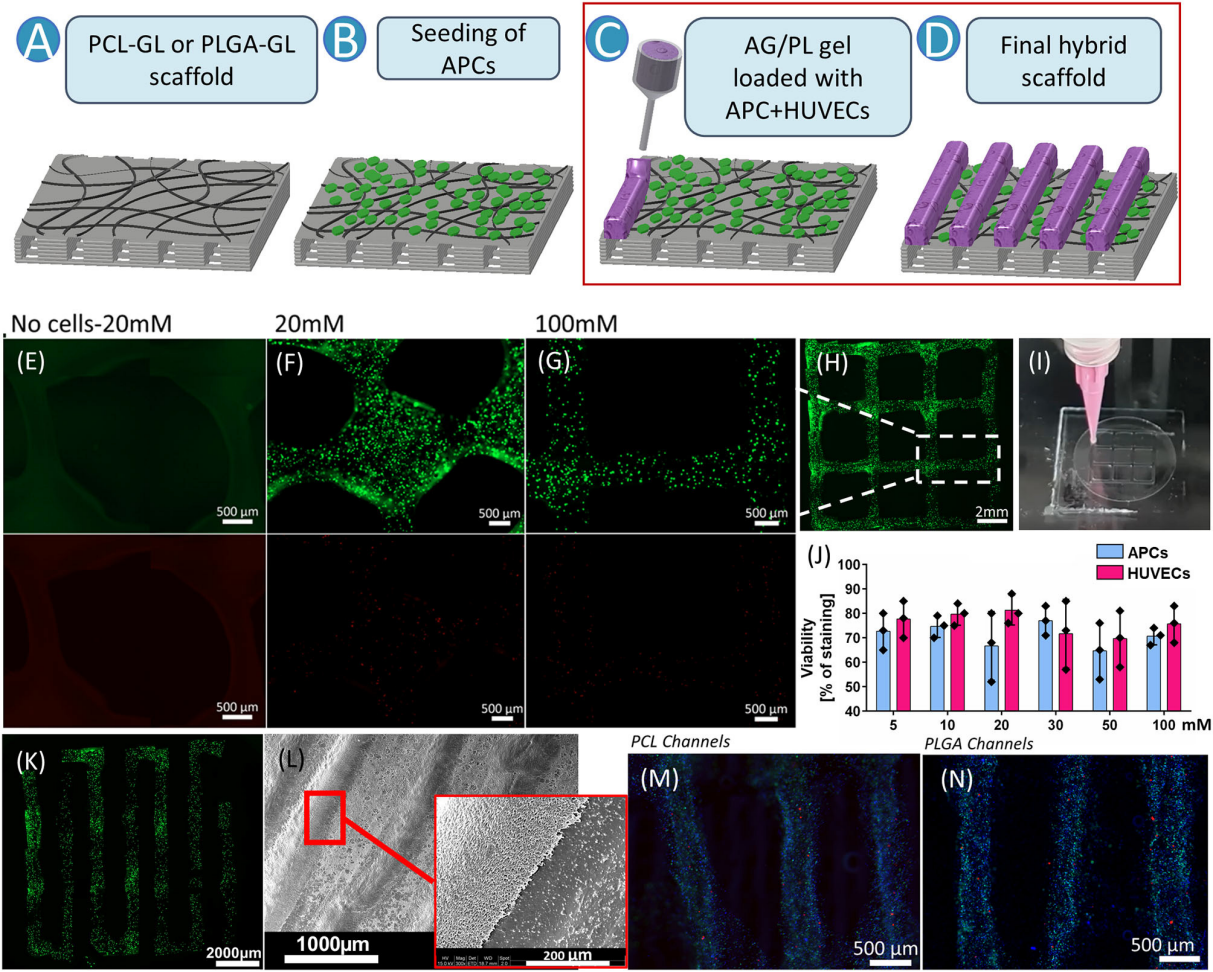
Effect of cross-linking on structure fidelity and cell viability. **(A–D)** Schematic representation of the final hybrid scaffold preparation following the specific steps (**A,B** were already described in Figure 3); **(C)** incorporation of APCs-HUVECs coculture in sodium alginate/Pluronic F127 gel (AG/PL); **(D)** hydrogel patterning on the seeded scaffold for the final assembling of the Hybrid scaffold. **(E,G)** show highly magnified fields of control (no cells) **(E)** 20 mM **(F)** and 100 mM **(G)** crosslinked gel; **(E)** shows the absence of unspecific stain in the gel without cells; **(F,G)** show the living cells stained with calcein (Green) while dead cells are stained with EthD-III (red); **(H)** Fluorescent microscopy image of the structure of the bioprinted structure in the shape of a 10 × 10 mm grid; Dotted square shows the magnified filed for **(G)**; **(I)** Picture of the 10 × 10 mm bioprinted grid; **(J)** Bar graph of cell viability of APCs and HUVECs loaded into the AG/PL gel and assessed after the exposure to different concentration of CaCl_2_. **(K)** Fluorescence image of the bioprinted structure of the final pattern for the hybrid Scaffold composition; **(L)** SEM picture of AG/PL gel printed onto the scaffold; **(M,N)** Representative images of the maintained pattern of the co-culture in the AG/PL gel extruded onto scaffold, after 5 days of incubation. APCs stained with Dil (red), HUVECs with DiO (green) and nuclei of both cell type with DAPI (blue). **(M)** poly(ε-caprolactone) with gelatin nanofibers coated with bioprinted sodium alginate/Pluronic F127 gel (PCL-GL + AG/PL) loaded with HUVECs + APCs; **(N)** polylactic-co-glycolic acid with gelatin nanofibers coated with bioprinted sodium alginate/Pluronic F127 gel (PLGA-GL + AG/PL) loaded with HUVECs + APCs.

Having evaluated the behavior of the APCs on the PCL-GL and PLGA-GL scaffolds, the final assembly of the hybrid scaffold consisted of the deposition of lines of gel, as described in Figures 6A,B. In fact, data shown above and results from a previous study (30), the polymeric scaffold itself did not ensure an ordered alignment of the cells. The spatial organization of the scaffolds was here enhanced by the deposition of gel with a specific pattern. The pattern of the scaffold improved the directionality of the cellularized structure (Figures 6C,D) aiming at promoting cells encapsulation and growth in specific lines.

The hydrogel was formed by a solution of AG due to its high biocompatibility and PL for its increased printability properties. Moreover, PL has the function of sacrificial material, when it is washed out it will generate bigger pores that improve cell interaction properties. AG and PL solutions were produced following the protocol described by Armstrong et al. (32). The final assembly of the hybrid scaffolds consisted of bioprinting the cell-laden gel to a specific pattern on the surface of the APC bioengineered PCL- and PLGA-based structures. The AG/PL gel was produced with a final concentration of 13% (w v^−1^) PL and 6% (w v^−1^) AG in serum-free DMEM.

Before incorporation into the final scaffold, the hydrogel underwent further fine-tuning of its properties. First, to improve the durability in wet conditions, the hydrogel was crosslinked with CaCl_2_ at different concentrations (5, 10, 20, 30, 50, 100 mM) (Figures 6E–G). Gel grids (12 × 12 mm) were bioprinted and, after 5 days of incubation in a culture medium, the morphology of the hydrogel structure was evaluated. Immunofluorescence images showed that grids incubated with a concentration of CaCl_2_ above 20 mM had better structure fidelity and maintained the bioprinted grid shape after 5 days of incubation (Figures 6H,I).

Next, we evaluated the possible cytotoxic effect of the crosslinking agent on cells loaded into the AG/PL gel. The analysis of cell viability showed that both APCs and HUVECs maintained viability (around 70%) through increasing concentrations of CaCl_2_ (Figure 6J). We selected 100 mM CaCl_2_ to use in subsequent studies, as this combined maintenance of scaffold shape with low cytotoxicity.

### Final Scaffold Cellularization: *In vitro* Evaluation of the Final Gel-Patterned Hybrid Scaffold Containing APCs and HUVECs Co-culture

The final assembly of the scaffold was performed using an AG/PL bioprinting pattern of a set of parallel lines along the (*x*) direction of the scaffolds to confer preferential direction of cell growth. The co-culture of APCs-HUVECs was used in the final hybrid scaffold to improve APCs action in promoting angiogenesis once implanted *in vivo*. The AG/PL gel was bioprinted with co-culture of APCs and HUVECs [at 1:4 ratio], with a total concentration of 4 million ml^−1^ cells. The shape gel lines were maintained for 5 days incubation (Figure 6K). From the observation of SEM images, the average line width of the extruded gel was 510 ± 33 μm and the adhesion of the AG/PL gel was confirmed (Figure 6L). Figures 6M,N illustrate higher magnification images of the complete hybrid scaffolds (PCL-GL + AG/PL and PLGA-GL + AG/PL), confirming the high preservation of the bioprinted morphology. This patterned scaffold prototype was then used in studies evaluating the potential of vascular engineering in a limb ischemia model.

### Perivascular Implantation of Cellularized Scaffolds Improves Collateralization and Accelerates Blood Flow Recovery

**Figure 7:**
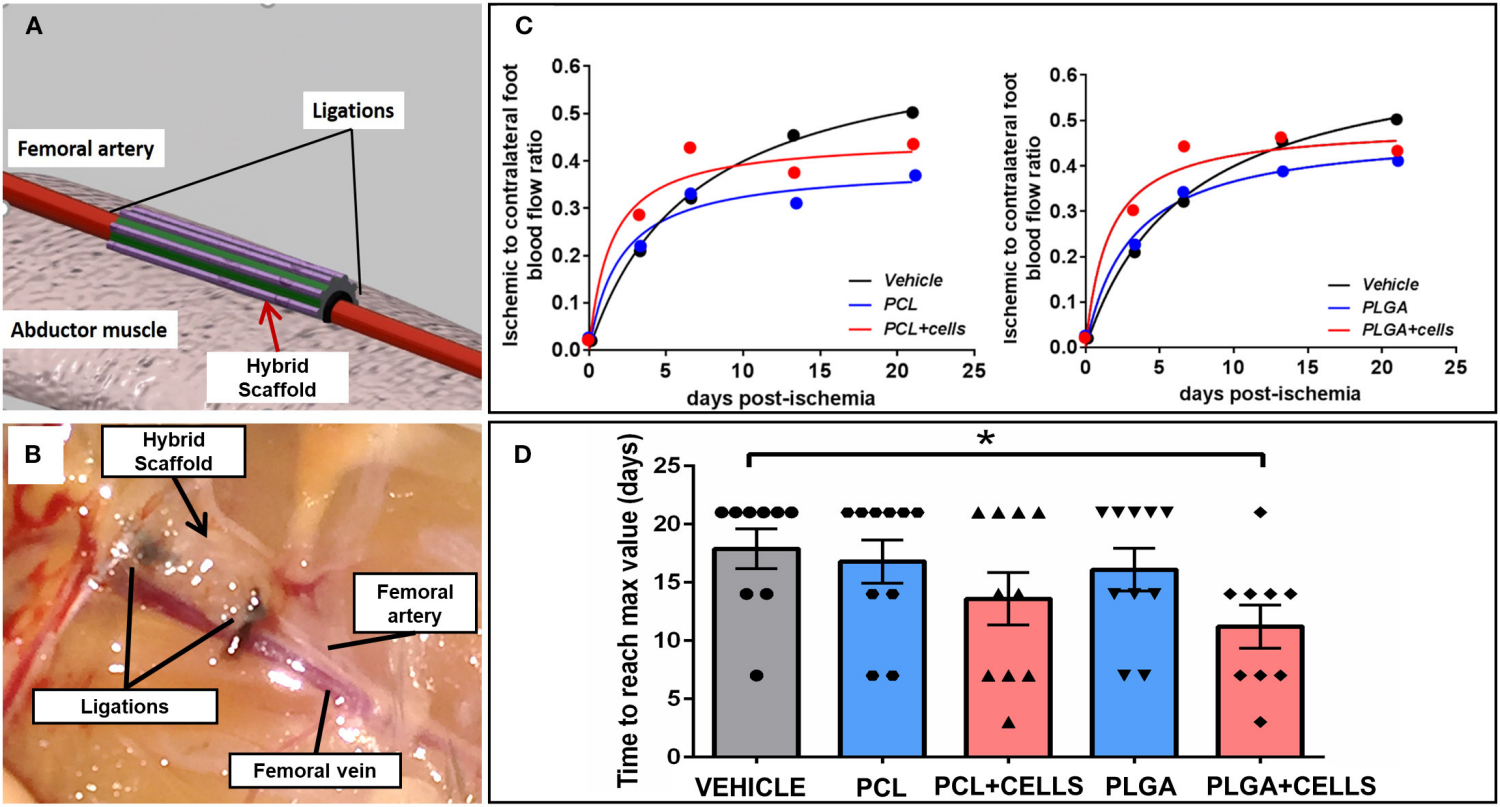
Outcomes of the *in vivo* implantation study. **(A,B)** Schematic representation **(A)** and photograph captured with optical microscopy of hybrid scaffold implantation **(B)**; **(C)** Time course of blood flow recovery expressed as the ratio of ischemic and contralateral limb; (*N* = 9–10 per group); **(D)** Bar graph showing the velocity of blood flow recovery; data expressed as mean ± SE; *N* = 9–10 per group. Groups: (1) Vehicle: induction of unilateral limb ischemia (LI) without treatment; (2) PCL: induction of LI and implantation of PCL-GL + AG/PL without cells; (3) PCL + CELLS: induction of LI and implantation of PLC-GL + AG/PL with cells; (4) PLGA: induction of LI and implantation of PLGA-GL + AG/PL without cells; (5) PLGA + CELLS: induction of LI and implantation of PLGA-GL + AG/PL with cells. *p < 0.05 vs. vehicle.

The revascularization capacity of the hybrid scaffolds was tested in a murine model of LI. Figures 7A,B shows the schematic approach of implantation and the anatomical site of LI induction. Five groups were studied: the control group did not receive any treatment, while the experimental groups were implanted with PLGA-GL + AG/PL or PCL-GL + AG/PL, with or without APCs/HUVECs. Analysis of blood flow recovery in the ischemic leg (normalized to contralateral side) showed that all groups reached a similar plateau (Figure 7C). However, calculation of the time necessary to reach the maximum recovery showed that the group implanted with cellularized PLGA-AG/PL recovered faster as compared with vehicle (11 vs. 18 days, respectively, *P* = 0.01). Likewise, the comparison between cellularized PLGA-GL + AG/PL and acellular PLGA-GL + AG/PL was also close to statistical significance (11 vs. 16 days, *P* = 0.07) (Figure 7D and Table 2). The comparison between cellularized PCL-GL + AG/PL and vehicle showed no significant difference in time to recovery (14 vs. 18 days, *P* = 0.015).

**Table 2:** Blood flow recovery.

**Group**	***n***	**Mean**	**95% Cl**
Vehicle	9	17.889	13.980–21.789
PCL	10	16.800	12.577–21.023
PCL + cells	10	13.600	8.472–18.728
PLGA	10	16.100	11.977–20.223
PLGA + cells	9	11.222	6.946–15.498

*Table showing the time for the groups to reach maximum blood flow recovery. Values are n of replicates per group, mean and the min and max values measured*.

**Figure 8:**
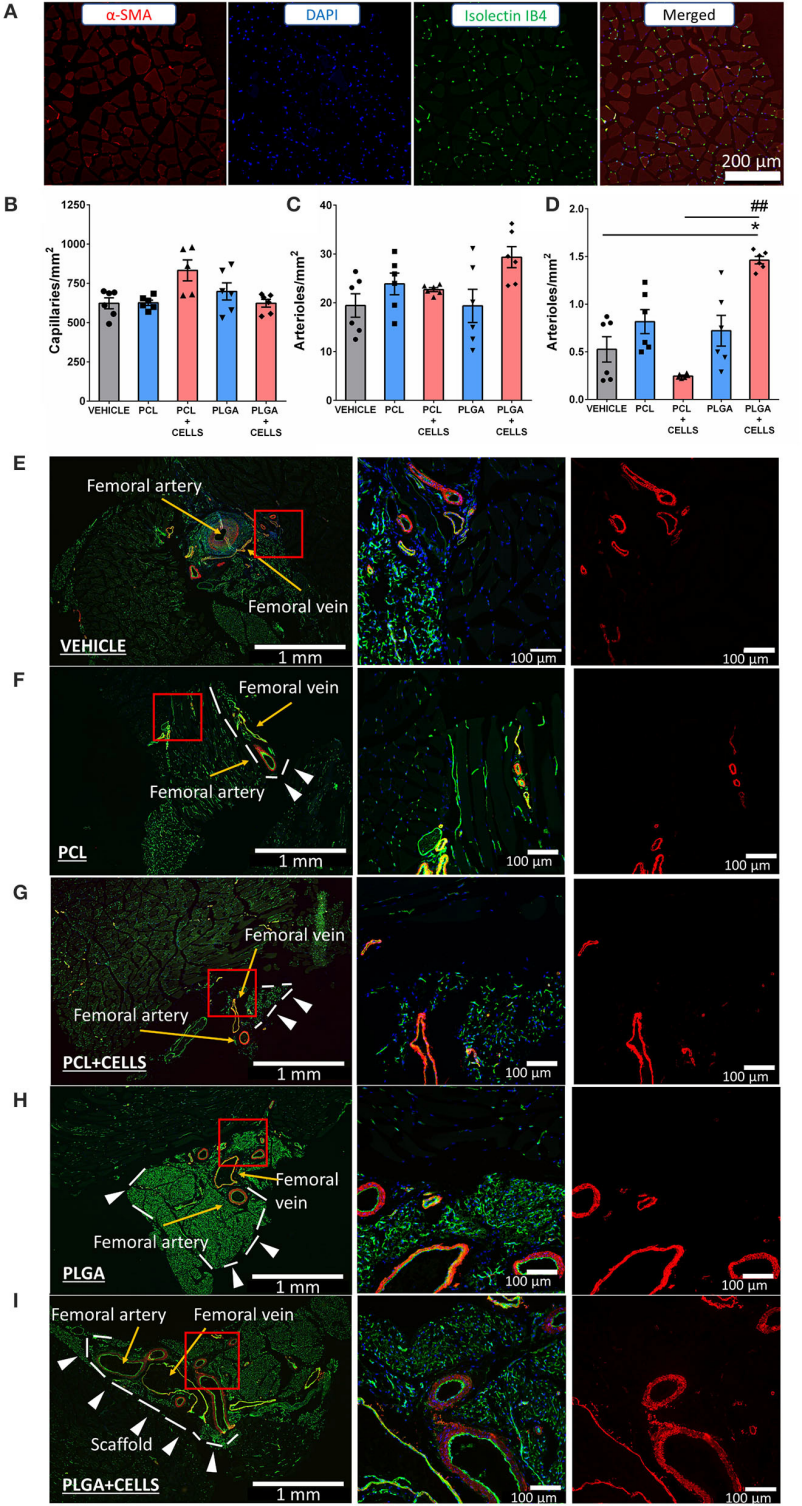
Angiogenesis assessment. **(A)** Representative fluorescent microscopy image of staining used to detect capillaries in the ischemic leg. **(B)** Bar graph showing capillary density; **(C,D)** Bar graphs of arterioles counting in the perivascular area: **(C)** cumulative calculation of arterioles density; **(D)** calculation of arterioles with diameter >50 μm; **(E–I)** Representative images of perivascular area of the various groups: Vehicle group **(E)**, PCL **(F)**, PCL + CELLS **(G)**, PLGA **(H)** and finally PLGA + CELLS group **(I)**. White arrows indicate the scaffold. Yellow arrows indicate arterioles. *P < 0.05 vs. vehicle. ^##^*P* < 0.01 vs. PCL-AG/PL without cells; data expressed in mean ± SE; N = 6 per group. Groups: (1) Vehicle: induction of unilateral limb ischemia (LI) without treatment; (2) PCL: induction of LI and implantation of PCL-GL + AG/PL without cells; (3) PCL + CELLS: induction of LI and implantation of PLC-GL + AG/PL with cells; (4) PLGA: induction of LI and implantation of PLGA-GL + AG/PL without cells; (5) PLGA + CELLS: induction of LI and implantation of PLGA-GL+AG/PL with cells.

Histological analysis of limb muscles revealed there was no difference in capillary density between groups (Figures 8A,B). Although the total number of arterioles was not different between groups (Figure 8C), narrowing the analysis to the arterioles with a diameter above 50 μm, animals given cellularized PLGA-GL + AG/PL scaffold showed a significant increase in this parameter compared with the vehicle group (*P* < 0.05) or those given cellularized PCL-GL + AG/PL scaffolds (*P* < 0.01) (Figure 8D). The increased collateralization induced in the mice with PLGA-GL + AG/PL scaffold may account for the accelerated perfusion recovery observed in the same group (Figures 8E–I).

## Discussion

A variety of techniques for by-passing total femoral artery occlusions and re-entering the true lumen in the distal artery are available, but few have been tested in randomized trials. The potentiation of collateralization represents a promising approach to provide relief to the ischemic tissue. In the present study, we attempted to achieve this goal through a tissue engineering approach, encompassing several innovative processes. First, we have set up a robust manufacture protocol for the hierarchical production of a hybrid scaffold made of bioprinted PCL and PLGA and electro-spun GL. Second, we verified the optimal combination of synthetic materials and natural products (GL) within the hybrid scaffold. Third, we gathered novel information regarding the ability of the scaffold to direct seeded APCs toward an angiogenic phenotype. Fourth, we succeeded in strengthening the directionality of the bioengineered scaffold by covering the initial layer of APCs with an additional patterned layer of AG/PL gel encapsulating a co-culture of APCs and HUVECs. Fifth, comparing two material formulations, with or without cells, in a murine model of femoral artery occlusion, we documented the superiority of cellularized PLGA-GL + AG/PL scaffolds in stimulating large arterioles formation around the blocked artery and encouraging faster blood flow recovery.

In a previous study, we set up an integrated manufacture protocol using a computer-assisted writing system to generate 3D scaffolds with woodpile or channel patterns and electro-spinning to deposit GL nanofibers onto the synthetic backbone. The structure was then engineered with human APCs to confer characteristics of a living material capable of supporting revascularization after perivascular implantation (30). Here, the successful approach was refined and extended, employing two synthetic materials, PCL and PLGA, as a backbone substrate. To achieve this goal, a piston-driven bioprinting system was employed; with results demonstrating versatility of the technique in achieving high-resolution and consistent geometry features (line width and thickness) for both PCL and PLGA synthetic matrices. We focused on a channel design because this patterning is the most appropriate to encourage alignment of new arterioles in limb muscles (30) and myocardial tissues (37–39).

Owing to differences in physical properties and biocompatibility, the choice of synthetic material is crucial for the success of medical scaffolds (40). Mechanical tests confirmed the anisotropy of the channel structures made of PCL and PLGA, confirming the more rigid behavior of the latter in line with previous literature (41, 42). Moreover, PCL maintained its stability over time, confirming the typical slow degradation rate in wet conditions; while PLGA revealed a more rapid decrease in both Young's Modulus and strain at rupture, which denotes greater fragility of the material.

Like ECM, scaffold micro- and nano-scale morphology (43) and biochemical cues can impact cell behavior (44, 45). Previous studies have explored the fabrication of composite scaffolds combining different manufacturing systems with promising results. This included rapid prototyping microfabrication techniques, like 3D printing, fusion deposition modeling and solid free form deposition, in combination with electrospinning to generate composite scaffolds (46, 47). In another study, electrohydrodynamic direct-jet was used to deposit microfibrous bundles of collagen-I in order to improve cell adhesion (48). The message from these studies was that composite scaffolds are better than scaffolds made of a single material in supporting cell viability. For this reason, and following on from our previous study (30), we improved our scaffolds by applying a layer of GL nanofibers using the electrospinning technique, so as to enhance the adhesion features of the synthetic backbone.

APCs are considered progenitors of different mesenchymal cell lines. Antigenic and expressional characterization of APCs demonstrated that these cells maintained their pericyte-like phenotype when seeded on 3D structures, as demonstrated by the expression of several transcription factors associated with stemness. This, together with the observed downregulation of fibroblastic markers, suggests that the 3D environment does not promote APC differentiation. Functional assays demonstrated an overall excellent biocompatibility of the studied materials. PCL- and PLGA-based scaffolds showed a similar capacity to support the viability of seeded APCs. Nonetheless, a molecular readout of apoptosis, the BAX/BLC2 ratio, was almost 2-fold higher in APCs seeded on PCL compared with APCs on PLGA, suggesting that the latter has superior biocompatibility. On the other hand, the PCL-based scaffolds improved cell adherence, which is compatible with PCL having mechanical properties closer to natural soft tissues (40). Previous studies showed that cell proliferation is greater on PCL than PLGA, due to the difference in stiffness of the two materials (40, 49, 50). We could not detect any difference in APC proliferation, possibly because of the presence of nanofibrous GL, which acts like a biomimetic substrate for growing cells.

Our previous transplantation study showed the complexity of molecular pathways implicated in APC-induced activation of neovascularization in a model of femoral artery occlusion (51). Here, we show that the APC proangiogenic profile is remarkably modified by the culture in a 3D environment, resulting in the induction of angiogenic (VEGFA) and arteriogenic (FGF) signaling. This expressional change could be advantageous in conditions requiring the formation of collateral vessels. It should be noted that *VEGFA* mRNA levels and VEGFA secretion in conditioned media showed similar increases in both cellularized 3D PLGA and PCL scaffolds compared with 2D control counterparts (from 4 to 6-fold, respectively). Meanwhile, ANGPT1 expression in conditioned media was downregulated in the 3D transition of PCL but not of PLGA. ANGPT1 is an oligomeric secreted glycoprotein that plays a key role in the organization and maturation of newly formed vessels, promoting the quiescence and structural integrity of adult vasculature (52).

A key advancement of this study is the use of a piston-driven bioprinting system to extrude a cell-laden gel containing a mixture of APCs and ECs onto the scaffold. Bioprinted AG/PL gel had less line width resolution when compared with the bioprinted synthetic polymer (PCL and PLGA). Nevertheless, the method was highly reproducible in depositing a series of parallel lines. The AG/PL gel lines would ideally synergize with the geometry of the PCL- and PLGA-GL channels in directing arterial collaterals in a parallel direction to the occluded femoral artery.

In the present study, we used two vascular cell populations to encourage collateralization, incorporating APCs in the initial layers and APCs and ECs into the patterned AG/PL gel. The advantage of combinatory administration is highlighted by previous successful studies using human MSCs and cardiac tissue-derived stem cells (CSCs) in a pig model of MI; with the combination showing superior results compared with a single cell population (53). Similarly, we have previously demonstrated that *in vivo* co-delivery of human CSCs and APCs reduced the infarct size and promoted vascular proliferation in a murine myocardial infarction model (10). One limitation of this study is the use of HUVECs instead of arterial ECs. However, this was proof of principle study, and specific EC populations could be used according to the implantation site.

After arterial occlusion, tissue recovery occurs through the opening of pre-existing collaterals and formation of new perivascular arterioles. Moreover, hypoxia induces the formation of new muscular capillaries. Results of hemodynamic and histologic analyses indicate the superiority of PLGA scaffolds in accelerating reperfusion and promoting the perivascular formation of arterioles with a diameter above 50 μm. Characteristics of the bio-engineered material and host response to the implant were identified as major players in regenerative processes activated by hybrid scaffolds.

The balance between mechanical stability and biodegradability play key roles in the design of successful therapies. Despite the PCL scaffolds having a better match of mechanical properties with the target tissue, the rate of degradation of the scaffold should be comparable to the growth of natural tissue (54). Here PLGA was demonstrated to have a much faster degradability, with a decay rate within our window of observation of 21 days. PLGA, also reportedly causes inflammation and robust angiogenesis (27), in contrast to the absence of inflammation and poor vascularization observed after the implantation of polyurethane and collagen-chitosan-hydroxyapatite (55). In addition, both PCL- and PLGA-based scaffold showed upregulation of pro-angiogenic factors, but the secretion of ANGPT1 was downregulated only in PCL scaffolds, possibly reflecting the lack of functional collateralization.

## Conclusion

Results of the present study represent an important step toward the clinical use of perivascular biomaterials for the revascularization around occluded limb arteries. Patients with diabetic vascular disease extending to regions below the knee could take advantage of this technique. We used xenogeneic human cells in immunocompetent mice to confirm the therapeutic efficacy of APCs in the absence of immunosuppression. This provides a scope for the use of allogeneic APCs in clinical trials. Furthermore, we have already upgraded the APC production using clinical-grade reagents, an important step forward clinical translation. However, additional improvements are necessary for scaling-up cell production and for integrating scaffold manufacture and bioengineering into a single process.

## Data Availability Statement

The raw data supporting the conclusions of this article will be made available by the authors, without undue reservation.

## Ethics Statement

The animal study was reviewed and approved by Guide for 307 the Care and Use of Laboratory Animals (The Institute of Laboratory Animal Resources, 1996) 308 under British Home Office PPL 30/3373.

## Author Contributions

PM and MC contributed to the development of the intellectual design of the project. MC designed and performed the main stages of the *in vitro* work. EJ, MF, TR, and BC contributed to the *in vitro* development of the research with different proportions. AT and MC designed the *in vivo* study and AT performed the animal work. AP and GV provided technical and intellectual support to the project. All authors contributed to manuscript revisions and read and approved the submitted version.

## Conflict of Interest

The authors declare that the research was conducted in the absence of any commercial or financial relationships that could be construed as a potential conflict of interest.
