# Supplementary material for: Fabrication of New Hybrid Scaffolds for in vivo Perivascular Application to Treat Limb Ischemia
Source: Front Cardiovasc Med. 2020 Nov 19;7:598890. doi: 10.3389/fcvm.2020.598890 (PMC7711071; doi:10.3389/fcvm.2020.598890)
Supplement: Supplementary file 1 [file Table_1.DOCX]

**Supplementary Information –**

**Fabrication of new hybrid scaffolds for *in vivo* perivascular application to treat limb ischemia.**

**Flow cytometry analysis**

APC suspensions were incubated with directly labeled antibody against for CD44, CD90, CD105, CD31 (Caltag Laboratories), CD45 (Miltenyi). Flow-cytometry analysis assessed on APCs showed the expression of the mesenchymal markers CD105 (72.1±18.1), CD44 (97.3±3.3), and CD90 (99.7±0.3), while they were negative for endothelial (CD31; 0.2±0.2) and hematopoietic (CD45; 0.4±0.3) antigens.

| **CELL LINE** | **Gender** | **Age** | **Experiment involved** |
| --- | --- | --- | --- |
| ***01.07.14E*** | M | 54 | *In vitro* functional assays / qPCR/ ELISA: biological replicate #1; |
| ***15.07.14D*** | M | 65 | *In vitro* functional assays / qPCR/ ELISA : biological replicate #3;  ***In vivo*** |
| ***09.06.16C*** | F | 77 | *In vitro* functional assays / qPCR/ ELISA: biological replicate #2; |
| ***08.07.16A*** | M | 85 | Bioprinting tuning and coculture: biological replicate #3; |
| ***15.07.16A*** | M | 71 | Bioprinting tuning and coculture: biological replicate #2; |
| ***26.07.16B*** | M | 70 | Bioprinting tuning and coculture: biological replicate #1; |

| **Table 1S: APCs isolated from donors were used for the specified experiments of the study.** |
| --- |
